# Glutathione peroxidase (GPX) activity in blood of ewes on farms in different scrapie categories in Iceland

**DOI:** 10.1186/1751-0147-50-23

**Published:** 2008-06-23

**Authors:** Kristín B Gudmundsdóttir, Jakob Kristinsson, Sigurdur Sigurdarson, Tryggvi Eiríksson, Torkell Jóhannesson

**Affiliations:** 1 Chief Veterinary Office, Section for Animal Diseases, Institute for Experimental Pathology, University of Iceland, Keldur v/Vesturlandsveg, 110 Reykjavík, Iceland; 2 Department of Pharmacology and Toxicology, Institute of Pharmacy, Pharmacology and Toxicology, University of Iceland, Hofsvallagata 53, 107 Reykjavík, Iceland; 3 Faculty of Natural Resources, Agricultural University of Iceland, Keldnaholt, 112 Reykjavík, Iceland; 4 Actavis Group, Clinical Research Department, Reykjavíkurvegur 80, 220 Hafnarfjördur, Iceland; 5 The Icelandic Food and Veterinary Authority, Austurvegur 64, 800 Selfoss, Iceland

## Abstract

**Background:**

Preliminary studies indicated decreased glutathione peroxidase (GPX) activity in blood of ewes on scrapie-afflicted farms. Other studies have shown decreased GPX activity in brain of prion-infected mice and in prion-infected cells *in vitro*. The aim of this study was to examine the GPX activity in blood as well as the distribution of GPX-activity levels from ewes on farms in scrapie-afflicted areas in Iceland.

**Methods:**

Blood samples were collected from 635 ewes (non-pregnant [n = 297] and pregnant [n = 338]) on 40 farms in scrapie-afflicted areas during the years 2001–2005, for analysis of GPX activity. The farms were divided into three categories: 1. *Scrapie-free farms *(n = 14); 2. *Scrapie-prone farms *(earlier scrapie-afflicted, restocked farms) (n = 12); 3. *Scrapie-afflicted farms *(n = 14). For comparison, 121 blood samples were also collected from non-pregnant ewes on one farm (farm A) in a scrapie-free area (scrapie never registered). Chi-square test was used to test for normal distribution of GPX-results, and Kruskal-Wallis test to compare GPX-results between categories.

**Results:**

The GPX-results appeared to be biphasically distributed in ewes in all three scrapie categories and on farm A. The presumptive breaking point was about 300 units g Hb^-1^. About 30–50% of the GPX-results from ewes in all three scrapie categories were below 300 units g Hb^-1 ^but only about 13% of the GPX-results from ewes on farm A. The mean GPX activity was highest on farm A, and was significantly lower on scrapie-prone farms than on scrapie-free or scrapie-afflicted farms (non-pregnant and pregnant ewes: P < 0.005, respectively; non-pregnant and pregnant ewes combined: P < 0.0005).

**Conclusions:**

1) the distribution of GPX-results in blood of Icelandic ewes apparently has a biphasic character; 2) the GPX-results were higher in ewes on one farm in a scrapie-free area than in ewes on farms in the scrapie-afflicted areas; 3) GPX-activity levels were significantly lowest on earlier scrapie-afflicted, restocked farms, which might have a bearing on the recurrence of sporadic scrapie on these farms; 4) further study on the possible role of GPX activity in the occurrence of scrapie in Iceland is warranted.

## Background

At least four forms of glutathione peroxidases (GPXs), containing selenocysteine as an active site, are found in the mammalian body. The best known of these is generally referred to as GPX-1 [1]. The activity of this isoenzyme has especially been studied in the blood of domestic animals (sheep, cattle, horses) and its activity may, with certain reservations, be taken as an indication of the selenium levels in the blood of the animals [2,3]. In the blood of sheep (and some other animals) more than 80% of the enzymic activity is confined to the cell membrane of the erythrocytes, but some activity can also be found in plasma [4]. In this text the acronym GPX (singular) is used to denote the GPX-1 form of the enzyme in the blood of sheep.

The activity of GPX results in the reduction of peroxides to water whether the peroxides are formed normally during metabolic exchange by the activity of superoxide dismutases or are the result of oxidative impact on the cells. Changes in GPX activity may accordingly affect the so-called antioxidative defense as well as have more subtle effects on cell activities and thus have a bearing on pathological processes in animals and man [1,5]. In this context, it is therefore of interest that decreased activity of glutathione peroxidase and superoxide dismutases in brain of mice experimentally infected with the pathological prion protein (PrP^sc^) also occur concomitantly, or prior to the development of neurological signs in the mice [6]. It is also noteworthy that reduced activities in these enzyme systems have been demonstrated in prion-infected hypothalamic cells resulting in increased susceptibility to oxidative stress [7].

In a preliminary study Jóhannesson et al. [5] found that the GPX activity was significantly lower in the blood of ewes on one scrapie-afflicted farm and two farms suspected of scrapie infection than in the blood of ewes on several scrapie-prone or scrapie-free farms. On the basis of a later study of ewes on farms in different scrapie categories Jóhannesson et al. [3] concluded that the generally low selenium concentration in sheep (cattle, horses) forage in Iceland is not likely to be directly connected with the occurrence of clinical scrapie but that it is still a matter of dispute whether the selenium concentration and GPX activity in blood of ewes on scrapie-prone or scrapie-afflicted farms are significantly different from ewes on scrapie-free farms. In the present study, conducted during the years 2001–2005, we therefore endeavoured to analyze the GPX activity in the blood of several hundreds of sheep on farms in different scrapie categories in order to substantiate whether the GPX activity or the distribution of individual results may relate to the occurrence, or recurrence, of clinical scrapie on the farms. Blood samples were, as far as possible, collected from non-pregnant ewes in the autumn as well as from pregnant ewes the following spring as the GPX activity normally varies with the concentration of selenium in the blood, from high levels in the autumn to low levels in the spring [3].

## Materials and methods

### Categories of farms, collection of blood samples and determination of GPX activity

**Figure 1:**
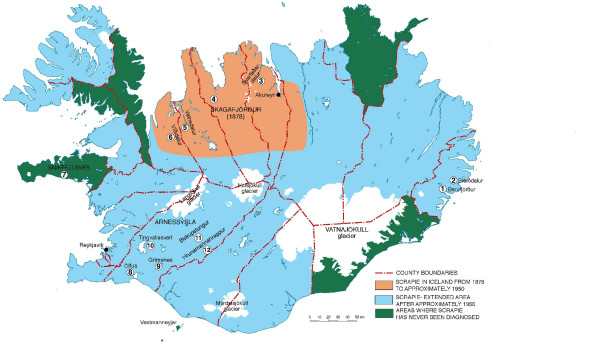
**Scrapie in Iceland and locations of farms**. Scrapie was, from its presumed origin in Skagafjörður in the year 1878, confined to a part of northern Iceland until ca. 1950 (orange). It has since spread patchily to greater or lesser parts of all counties (blue) except for four (green). The numbers 1–12 indicate the twelve different locations of the 41 farms in all scrapie categories where 756 blood samples were collected for the analysis of GPX activity. Farm A is located in the green county (Snæfellsnes) numbered 7. The scrapie-free counties and the large green area in the north-east corner of the country are the main areas in the country used to provide healthy lambs to restock formerly scrapie-afflicted farms.

Blood samples were collected from 635 ewes on 40 farms in scrapie-afflicted areas during the years 2001–2005. The farms were divided into three categories according to scrapie status: 1. *Scrapie-free farms*: 14 farms where scrapie has never been diagnosed or diagnosed prior to 1960 and then restocked with healthy sheep. 2. *Scrapie-prone farms*: 12 farms afflicted by scrapie after 1980 and afterwards restocked with healthy sheep. 3. *Scrapie-afflicted farms*: 14 farms where scrapie was diagnosed during the research period (2001–2005). The locations of the farms are shown in Fig. 1. It can be seen from the figure that farms in the three scrapie categories are located amongst each other in the areas depicted as orange or blue in the map.

The blood samples were collected from 2–5 year old ewes. All the ewes were examined by a veterinarian before the sampling. None of them showed any signs of clinical disease. On 20 of the farms, blood samples were collected from both non-pregnant ewes in the last trimester of 2002, shortly after the sheep had been gathered from the mountain pastures, and from pregnant ewes in the first half of 2003 when the ewes had been housed and fed inside for months. On the remaining 20 farms (including the 14 scrapie-afflicted farms), blood samples were collected either from pregnant or non-pregnant ewes. All sheep on the scrapie-afflicted farms were, according to government regulations, culled shortly after the disease had been diagnosed (usually one or a few animals in a flock), and the farms were placed in quarantine. These rules in fact preclude the study of any large groups of ewes diagnosed with clinical scrapie. In total 297 samples were collected from non-pregnant ewes and 338 from pregnant ewes (Table 1). The total number of results in the three scrapie categories also includes some results of GPX analyses from northern Iceland (orange, Fig. 1) that have previously been published by Jóhannesson et al. [3,5].

**Table 1:** Number of blood samples collected from non-pregnant and pregnant ewes on 40 farms in the three scrapie categories in scrapie-afflicted areas (see also text).

**Category**	**No of farms**	**No of samples from non-pregnant ewes**	**No of samples from pregnant ewes**	**Total no of samples**
*Scrapie-free farms*	14	94	113	207
*Scrapie-prone farms*	12	120	119	239
*Scrapie-afflicted farms*	14	83	106	189
Total numbers*	40	297	338	635

*: Total number of farms, total number of samples from non-pregnant and pregnant ewes, respectively, and grand total of samples.

In order to get an estimate of the levels and distribution of individual values of GPX activity in the blood of ewes in one particular sheep flock, blood samples were also collected from 121 two-five year old ewes on a sheep farm (referred to in the text as farm A) in an area where scrapie has never been diagnosed (green, Fig. 1). These samples were collected from non-pregnant ewes in the autumn of 2005. This farm (and neighbouring farms) has been used for decades to provide healthy lambs to restock scrapie-afflicted farms after expiration of the statutory quarantine period and thus qualifying them as scrapie-prone farms (cf. above).

The blood samples were drawn from the jugular vein into 9 ml tubes containing lithium-heparin as an anticoagulant (Sarstedt, Nümbrecht, Germany). The samples were refrigerated as soon as possible and they were analysed within 48 hours. GPX activity in whole blood was determined with a spectrophotometric assay at the Institute for Experimental Pathology, University of Iceland, Keldur, Reykjavík, as described by Jóhannesson et al. [3]. The coefficient of variation (C.V.) for the method was consistently around 8% when tested on several occasions on blood samples obtained from the stock of sheep kept at the Keldur Institute during the five year span of the study. The results are expressed as units g Hb^-1^. All the ewes had hemoglobin (Hb) levels within the normal range (90–145 g l^-1^).

### Statistical analysis

Chi-square test was used to test for normal distribution of GPX-activities in the blood of ewes on farms in the three scrapie categories as well as on farm A. Kruskal-Wallis test was used to compare GPX-activities between categories.

## Results

**Figure 2:**
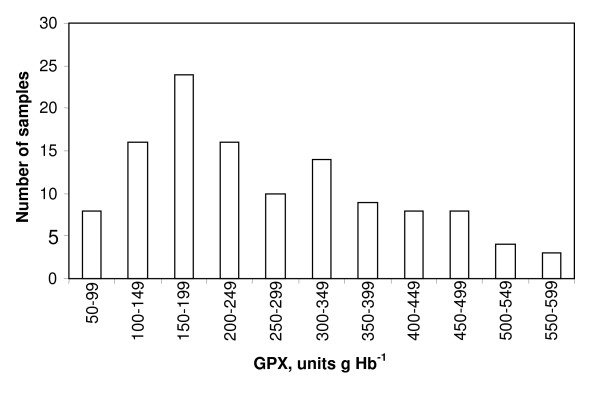
**The distribution of individual analyses of GPX activities in non-pregnant ewes on scrapie-prone farms**. The results were apparently biphasically distributed, with a marked relative dominance of low individual results (lower than 300 units g Hb^-1^).

**Figure 3:**
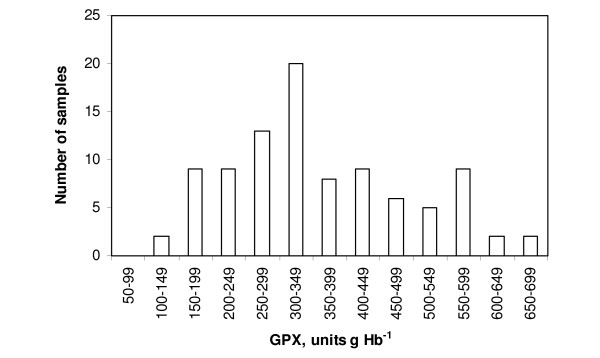
**The distribution of individual analyses of GPX activities in non-pregnant ewes on scrapie-free farms**. The results were apparently biphasically distributed, with a relative dominance of high individual results (higher than 300 units g Hb^-1^).

**Figure 4:**
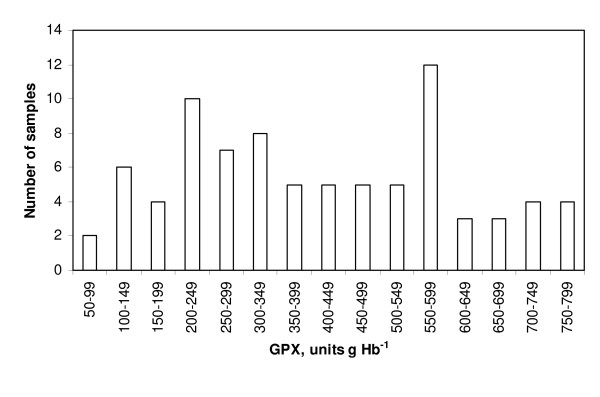
**The distribution of individual analyses of GPX activities in non-pregnant ewes on scrapie-afflicted farms**. The results were apparently biphasically distributed, with a relative dominance of high individual results (higher than 300 units g Hb^-1^).

**Figure 5:**
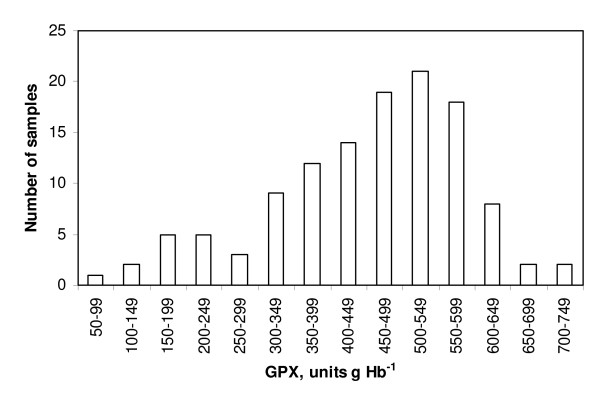
**The distribution of individual analyses of GPX activities in non-pregnant ewes on farm A**. The results were apparently biphasically distributed, with a marked relative dominance of high individual results (higher than 300 units g Hb^-1^).

The results are summarized in Figures 2, 3, 4, 5 and Table 2.

**Table 2:** The means, medians and the range of individual results of GPX determinations (units g Hb^-1^) in blood from non-pregnant and pregnant ewes on farms in the three scrapie categories in scrapie-afflicted areas (see also text) and on farm A.

	**Scrapie-free farms**	**Scrapie-prone farms**	**Scrapie-afflicted farms**	Farm A*
**Non-pregnant ewes**				

Means	312	267	361	452
Medians	280	243	342	478
Range	92–625	67–585	36–733	94–707

**Pregnant ewes**				

Means	298	239	278	-
Medians	267	189	240	-
Range	71–625	58–625	54–766	-

*: The ewes on farm A were all non-pregnant.

When estimated with the chi-square test, the GPX results from ewes in the three scrapie categories were not found to be normally distributed, except for the GPX results from non-pregnant ewes on scrapie-afflicted farms. Subsequent statistical comparison between categories was therefore carried out by the use of a non-parametrical test (Kruskal – Wallis test). The results of GPX determinations in the ewes on farm A were also normally distributed by the chi-square test.

By sight the GPX results were distributed more or less biphasically in ewes (non-pregnant or pregnant) in all three scrapie categories and also on farm A. The presumptive breaking point was about 300 units g Hb^-1^. More than half of the GPX results from non-pregnant ewes on the scrapie-prone farms were below 300 units (64 out of 120 [about 53%], Fig. 2). In non-pregnant ewes on scrapie-free and scrapie-afflicted farms the corresponding percentage figures were 35.5% and 37%, respectively (Figs. 3 and 4). Results for the pregnant ewes were also more or less biphasically distributed and the percentage numbers were of the same order as for the non-pregnant ewes in all three scrapie categories. In the ewes on farm A only relatively few GPX results were below 300 units g Hb^-1 ^(16 out of 121 [about 13%], Fig. 5). The marked dominance of high GPX results in ewes on farm A is also borne out by the fact that the median value was higher than the mean value, whereas for ewes in the three scrapie categories the median value was in every case lower than the mean value (Table 2).

The mean GPX activity was highest by far in the non-pregnant ewes on farm A (Table 2). The mean GPX activity was significantly lower in non-pregnant and pregnant ewes, respectively, on scrapie-prone farms than on scrapie-free farms or on scrapie-afflicted farms (P < 0.005, respectively). When the whole groups, comprising both non-pregnant and pregnant ewes in these categories, were compared statistically, the difference was even more marked (P < 0.0005). Significant difference was not found between the mean GPX activities from ewes on scrapie-free farms and scrapie-afflicted farms (P > 0.05, Table 2).

The mean GPX activity in non-pregnant ewes in all three scrapie categories combined was significantly higher than in the pregnant ewes in these categories (P = 0.0027).

The GPX results varied 6–11 fold in ewes on scrapie-free farms and scrapie-prone farms as well as on farm A, and between 14–20 fold in ewes on the scrapie-afflicted farms (Table 2).

## Discussion

It should be noted that the term "scrapie-prone" as used in this text refers especially to the fact that in recent years many cases of scrapie have been observed sporadically on casual farms where scrapie had been diagnosed previously, the flocks culled and the farms subsequently restocked with healthy sheep in accordance with government rules. It should also be noted that information on the occurrence of scrapie is in general fragmentary before 1960 and that systematic, preventive measures against scrapie (including the culling of flocks, quarantine periods etc.) were first legally enforced just prior to 1980. Thus these two years have been used as cut-out times in this study (cf. Materials and methods).

The scrapie-prone and scrapie-afflicted farms were, with two exceptions (no.1 and 2, Fig. 1), located in two large areas, one in the northern part of the country (scrapie first diagnosed before 1950) and the other in the the southern part (scrapie first diagnosed after 1950), where scrapie has repeatedly been registered (no. 3–6 and 8–12, respectively, Fig. 1). The scrapie-free farms were also located in these two areas. Thus, the study of ewes on farms in the three scrapie categories is in essence based on analyses of GPX activity on farms in two scrapie-afflicted areas that are geographically distinct and also different with respect to the history of scrapie. The results may therefore be considered as representative of the GPX activity in ewes living in scrapie-afflicted areas in Iceland.

Whether or not normally distributed according to the chi-square test, the GPX results were apparently biphasically distributed in all scrapie categories, and also on farm A. The presumptive breaking point was about 300 units g Hb^-1 ^(Figs. 2, 3, 4, 5). The biphasic distribution was, however, more conspicuous in the scrapie-prone ewes (Fig. 2) and the ewes on farm A (Fig. 5) than in the ewes on farms in the other scrapie categories (Figs 3, 4). Biphasic distribution of individual GPX results in blood of sheep has been described in Northern Ireland and Finland (8, 9, 10). These authors considered the biphasic distribution to be genetically determined. In their studies low GPX activity was reciprocated in low selenium concentration in the blood and high GPX activity in high selenium concentration. Thus in a given flock of sheep there should be some individuals that are likely to have innate low GPX activity with low selenium concentration in the blood and at the same time other individuals that are likely to have high GPX activity with high selenium concentration in the blood.

The shift from the dominance of relatively many high GPX results in ewes on the scrapie-free farm A to the relatively fewer high GPX results in sheep on farms in the three scrapie categories in the scrapie-afflicted areas is perhaps best explained by different levels of selenium in the blood of the ewes. This may especially apply to the low levels of GPX activity in ewes on the scrapie-prone farms (see below). To our knowledge selenium has not been studied in sheep forage or vegetation on sheep pastures in the Snæfellsnes County (Fig. 1) and the previous study of Jóhannesson et al. [3] on selenium and GPX activity was confined to farms in scrapie-afflicted areas. The possibility therefore exists that the selenium concentration is in fact higher in the sheep pastures in scrapie-free areas than in the scrapie-afflicted areas. This is obviously a topic for further studies. It should also be mentioned that GPX results from another study in ewes on two farms in the neighbourhood of farm A were found to be in the same range as the results from ewes on this farm (unpublished results).

High GPX activity in the ewes on farms in the scrapie-free areas might also be linked to other trace elements than selenium. Zinc appears to be a cofactor to glutathione peroxidases and this trace element (along with manganese) has been shown to occur in significantly higher concentrations in sheep forage on farms in scrapie-free areas than in forage on farms in scrapie-afflicted areas [11,12]. High concentration of soluble iron in the soil, as is often found in Iceland [13], may efficiently compete with zinc for absorption in the roots of plants. It is therefore noteworthy that the lowest iron concentration is found in the forage on farms in scrapie-free areas where the zinc concentration is at the highest [11,14].

The mean GPX results were significantly lower in the blood of non-pregnant and pregnant ewes, respectively, on scrapie-prone farms than in non-pregnant and pregnant ewes on scrapie-free farms and on scrapie-afflicted farms (Table 2). There is no simple explanation to these results and especially as the difference was observed at both high selenium concentration in the blood (non-pregnant) and low selenium concentration (pregnant) [3]. Andrés et al. [15] ascribed low GPX results in lambs especially to low levels of sulphur in the soil. This does not seemingly apply to our findings as the sulphur content in sheep forage has been found almost the same all over the country [16]. A possible explanation could be that newly acquired sheep, often relatively few in number, on earlier scrapie-afflicted farms (the scrapie-prone farms) are kept more close to the farms, and are not until later dispersed to the mountain pastures to the same extent as the flocks of sheep in the other categories. The explanation might thus be that the sheep on the scrapie-prone farms have, during summer time, in general been grazing on grasses containing less selenium than is found in the highland vegetation (cf. 3).

This idea is in fact supported, in part at least, by the results of Jóhannesson et al. [3]. These authors found that both the selenium concentration in blood and the GPX activity was higher in non-pregnant ewes on three scrapie-free farms located in the Vatnsdalur Valley than on five scrapie-prone farms in the same area (no. 5, Fig. 1). However, the results from the pregnant ewes on farms in the two scrapie categories did not differ significantly in this study. It should be noted that these GPX results from northern Iceland are included with the other results in the present study (cf. Materials and methods).

Decreased glutathione peroxidase and superoxide dismutase activity in brain of prion-infected mice and prion-infected cells *in vitro *have, as mentioned, been seen to precede or accompany the clinical signs of experimental infection in the animals on the one hand and weaken the resistence of the infected cells to reactive oxygen species on the other hand [6,7]. Thus it seems possible that low GPX or superoxide dismutase activity could, due to defective antioxidative defense or otherwise, hasten the recurrence of scrapie in sheep newly brought to scrapie-prone farms where the infectious prion protein may loom for years in the soil or elsewhere in the environment [17,18]. As many cases of scrapie have been diagnosed sporadically during recent years on scrapie-prone farms the subject clearly justifies further study. In this context it should be noted that the results of a preliminary study of Jóhannesson et al. [5] apparently indicated that superoxide dismutase (SOD1) activity in erythrocytes was lower in sheep on scrapie-prone farms than in sheep on farms in the other scrapie categories. However, this finding could not be substantiated in a later and more thoroughly performed study [19].

The average GPX activity was significantly lower in the pregnant ewes in the spring than in the non-pregnant ewes in the autumn shortly after they had been gathered from the mountain pastures. This is in accordance with the previous findings of Eiríksdóttir et al. [2] and Jóhannesson et al. [3]. These authors also found that the selenium concentration in blood was significantly correlated to GPX activity although Jóhannesson et al. [3] found this correlation much less pronounced in pregnant than non-pregnant ewes. GPX activity may thus be used as an indicator of the selenium concentration in the blood of sheep when comparing groups of animals, but preferably only in non-pregnant animals. However, due to the more or less biphasic distribution of GPX results and the concomitant wide variability of the individual results, especially observed on scrapie-afflicted farms (Table 2), the use of GPX activity as an indicator of the selenium concentration in the blood of sheep should always be planned with great care.

It was concluded from this study that: 1) the distribution of GPX results in the blood of Icelandic ewes apparently has a biphasic character; 2) the GPX-activity levels were higher in ewes on one farm in a scrapie-free area than in ewes on farms in three scrapie categories in the scrapie-afflicted areas; 3) GPX-activity levels were significantly lowest on earlier scrapie-afflicted, restocked farms, which might have a bearing on the recurrence of sporadic scrapie on these farms; 4) further study on the possible role of GPX activity in the recurrence, or occurrence, of scrapie in Iceland is warranted; 5) the use of GPX activity as an indicator of the selenium concentration in the blood of sheep should be planned with great scrutiny.

## Authors' contributions

All authors contributed equally to the research. All authors read and approved the final manuscript.
